# Supplementary material for: Aperiodic neural timescales in prefrontal cortex dilate with increased task abstraction
Source: bioRxiv. 2025 May 13:2025.04.21.649913. Preprint. [Version 2] doi: 10.1101/2025.04.21.649913 (PMC12132356; doi:10.1101/2025.04.21.649913)
Supplement: Supplementary file 1 [file NIHPP2025.04.21.649913v2-supplement-1.pdf]

SUPPLEMENTARY FIGURE 1

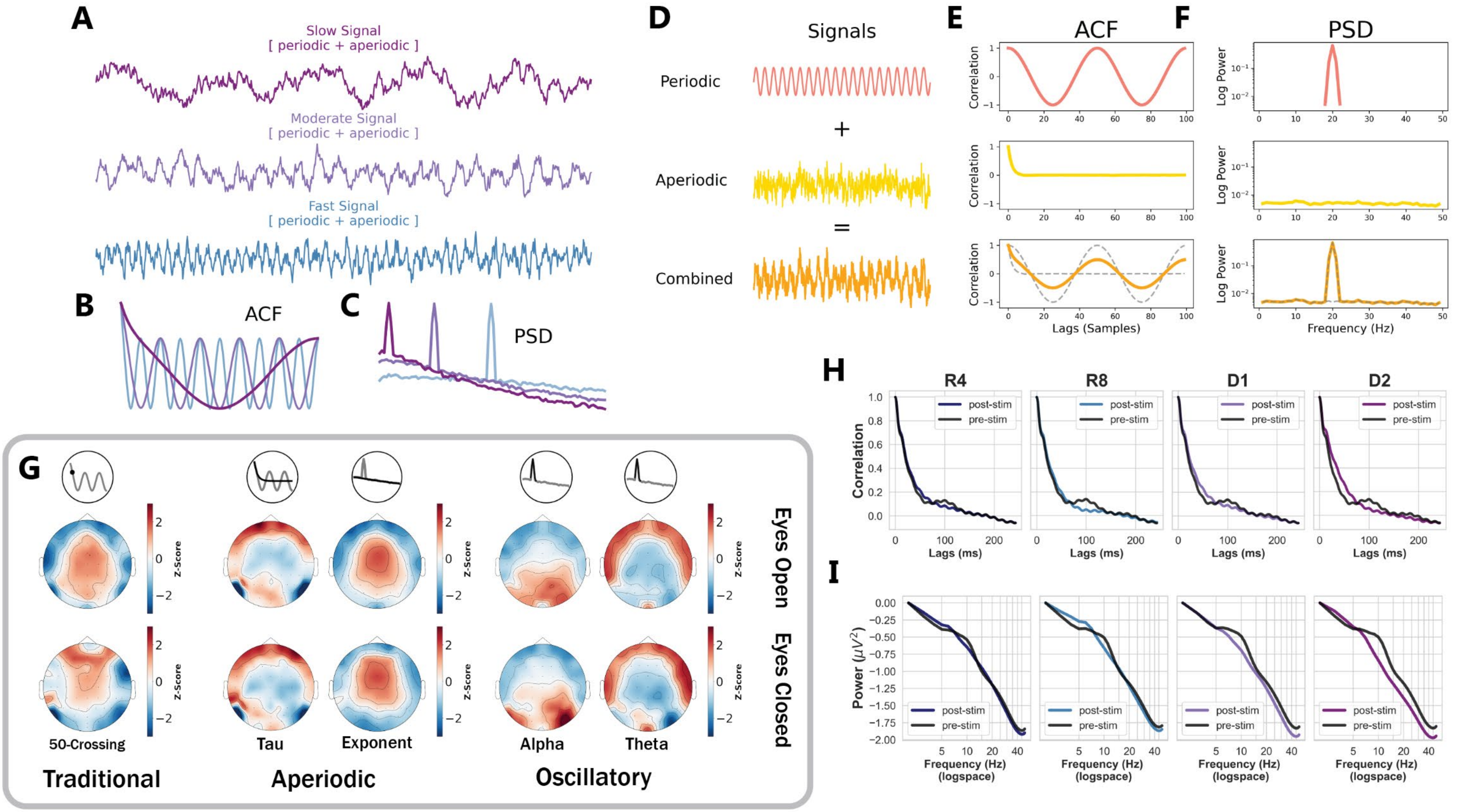

Figure S1: Neuronal timescales can be measured in either the Autocorrelation Function (ACF) or Power Spectral Density (PSD) space:

A) Simulated signals with rhythmic (oscillatory) and non-rhythmic (aperiodic) components, generated to show 3 different “levels” of low-to-high frequency activity. Neural signals can vary in terms of their overall composition of stable, low-frequency, and dynamic, high-frequency signals.

B) An autocorrelation function can be conceptualized as describing the rate of decay in the self-similarity of a timeseries signal. Signals that are “faster,” or closer to white noise, result in ACFs that decay quickly. Conversely, signals that are “slower,” or closer to pink/brown noise, result in ACFs that take more lags to decay (extending out further into the x-axis). Oscillations in the time domain of a signal manifest as oscillatory-like activity in the ACF, and likewise reflect the frequency of the oscillation.

C) The power spectral density plots of the three simulated signals in A. Signals closer to white noise manifest in the PSD as flatter spectra, while the slowest signal yields a steeper spectrum. Oscillations appear as peaks over and above the “background” of the aperiodic activity. The knee of the aperiodic component of the power spectrum can be used to mathematically approximate the decay rate of the ACF.

D) Simulated signals: a 20 Hz sine wave (red, top) combined with aperiodic activity (yellow, middle) produces a signal (orange, bottom) that mimics neural data in its combination of rhythmic and non-rhythmic signals.

E) and F) The respective autocorrelation functions (ACF) and power spectral densities (PSD) resulting from transformations of the corresponding signals in A. While a pure sine wave in the time domain appears similarly rhythmic in the ACF space (E, top), it appears as a peak in the PSD space centered at the oscillatory frequency (F, top). The combination of oscillatory and aperiodic signals produces ACFs and PSDs (E and F, bottom) that resemble those derived from neural data.

G) Distribution of each neural metric (from left to right: 50-Crossing, ACF-Tau, PSD-Exponent, PSD-Alpha bandpower, and PSD-Theta bandpower) over electrodes during eyes-open and eyes-closed resting state, from Dataset 1.

H) Subject and trial-averaged raw ACFs for the pre- and post-stimulus windows, for each condition. The high-abstraction, high-difficulty D2 condition shows visibly slower timescales that manifest as longer decays in the ACF.

I) Same as H, but showing raw PSDs plotted with offset-correction to align all spectra to zero. PSD knees at lower frequencies correspond to slower timescales. Averaging across subjects and trials may also distort oscillatory peaks in the underlying data, necessitating parameterization at a more granular level.

SUPPLEMENTARY FIGURE 2

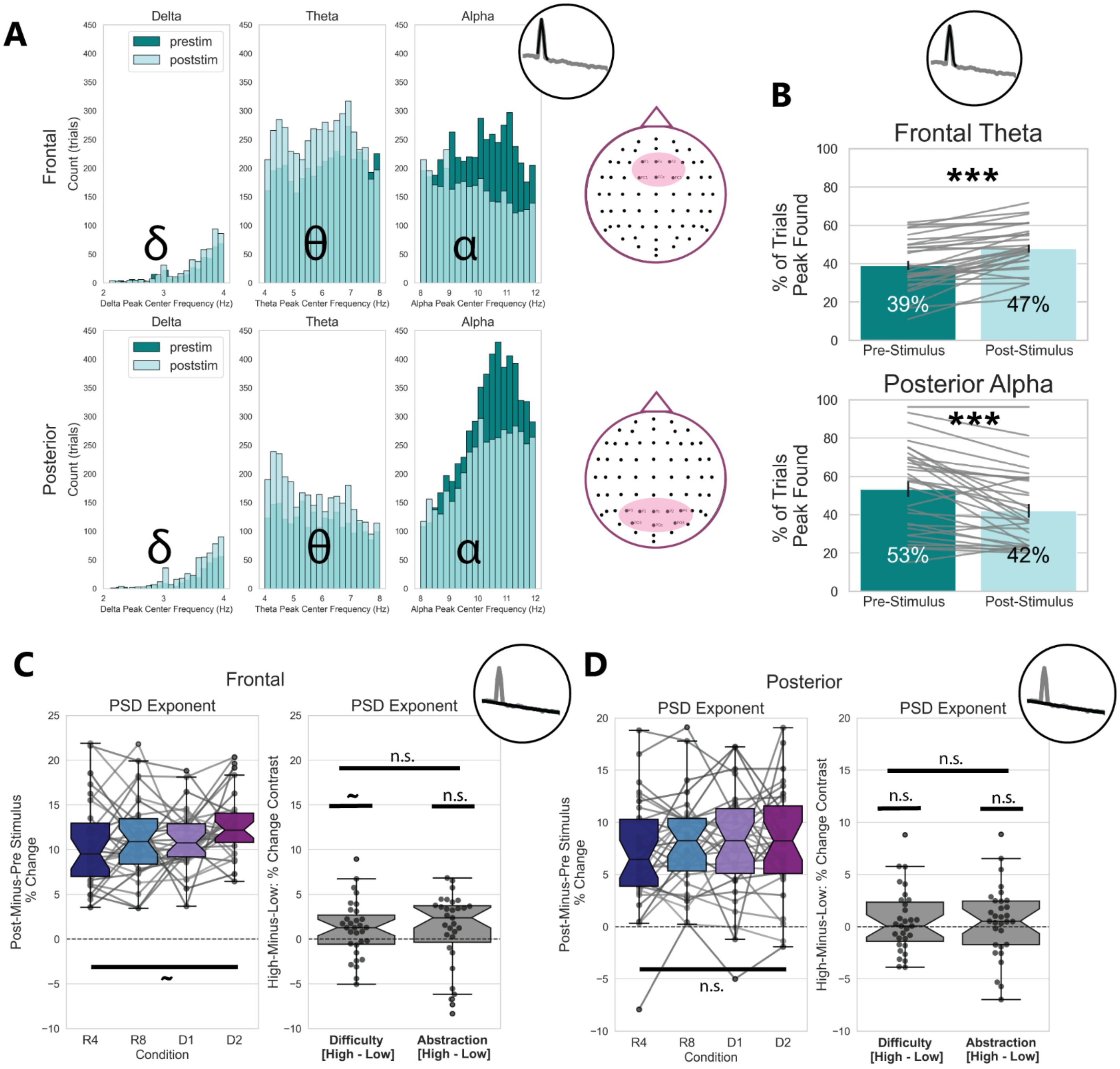

Figure S2: Oscillatory Peak Presence Differs between Pre-and-Post Stimulus Time Windows

A) Histograms of oscillatory peaks detected from PSD Oscillatory Peak model fitting, pooled across all subjects, all conditions, and all trials. Oscillatory peaks detected in the theta range (4-8 Hz) in Frontal Channels (top) are more numerous after stimulus onset, while oscillatory peaks detected in the alpha range (8-12 Hz) in Posterior Channels (bottom) decrease after stimulus onset. Model fits of delta oscillatory peaks at the lower bound edge of the power spectrum (2 Hz) are considered artifactual and were removed from plotting here.

B) Same as A, but plotted in terms of the percent of all trials. PSD parameterization only successfully detected oscillatory peaks in a maximum of 53% of trials (pre-stimulus Alpha peaks), and the numbers of detected peaks shifts pre-to-post stimulus for both theta and alpha peaks. A McNemar test for significance revealed a significant effect of time window and oscillation type on oscillatory peak presence.

C) and D) The trial-averaged aperiodic measure PSD-Exponent from Frontal and Posterior channels did not vary significantly across conditions, though it was trending ($p = 0.076$) in Frontal channels. They did not show a main effect of either Difficulty or Abstraction.

SUPPLEMENTARY FIGURE 3

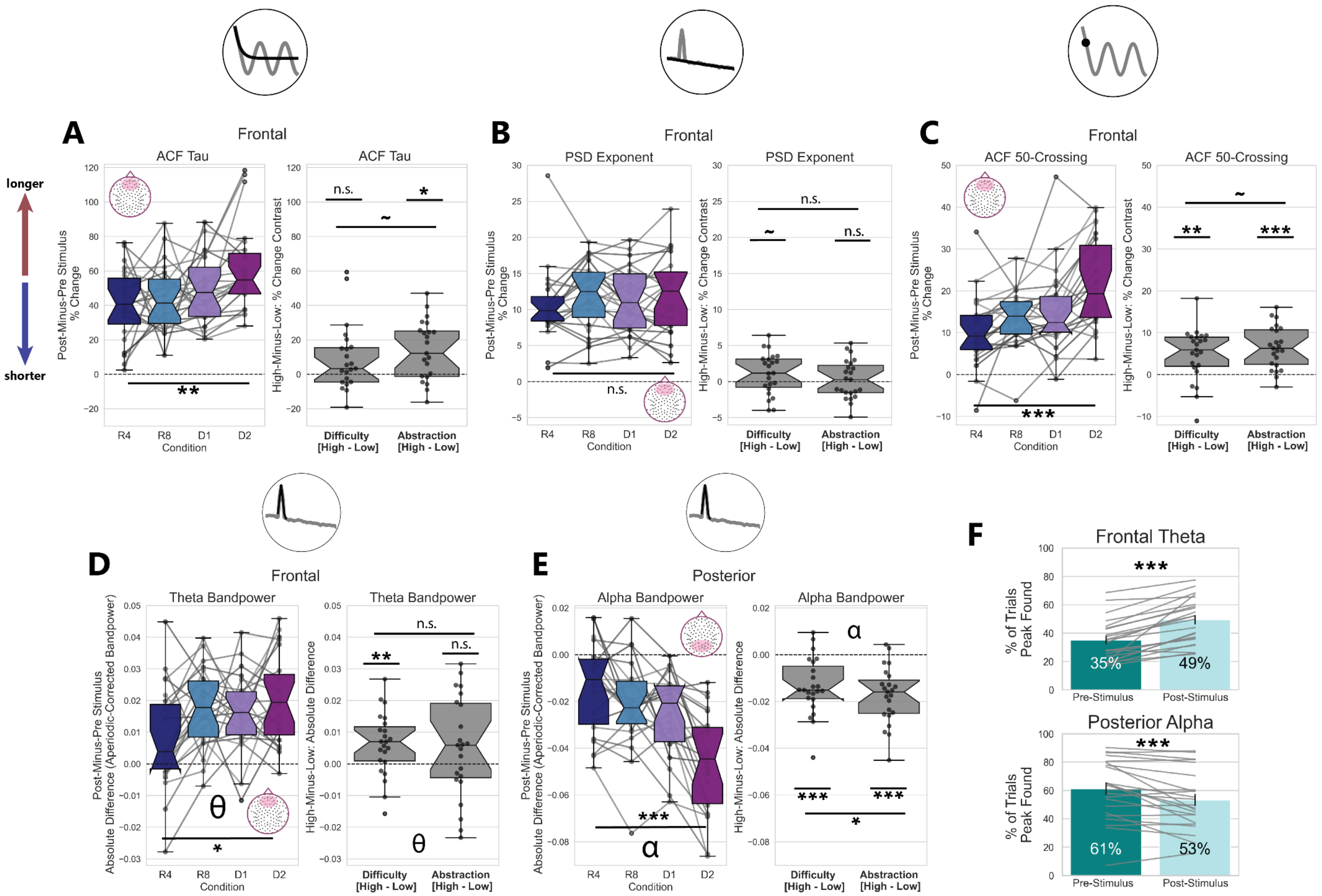

Figure S3: Aperiodic and oscillatory timescales lengthening from pre-to-post stimulus replicated in independent dataset

A) The ACF Tau parameter measured at Frontal electrodes in Dataset 2 exhibits a condition-specific increase pre-to-post stimulus. This finding replicated for the main effect of Abstraction, but not for the Abstraction and Difficulty interaction.

B) The Frontal PSD Exponent did increase post-stimulus relative to pre-stimulus, but this increase did not vary significantly across conditions.

C) The Frontal 50-Crossing measure from the ACF exhibited a significant increase, with a main effect of condition, Abstraction, and Difficulty. These data replicate the previous dataset, with the exception of finding only a trending interaction between Abstraction and Difficulty.

D) and E) Frontal aperiodic-corrected theta bandpower replicated the previously observed main effect of condition, and a main effect of Difficulty, but not the main effect of Abstraction. The previous findings of posterior aperiodic-corrected alpha bandpower decrease, with main effects of condition, Abstraction, and Difficulty, as well as an interaction between Abstraction and Difficulty were replicated.

F) *In line with findings from Dataset 1, the number of theta oscillatory peaks detected by PSD parameterization decreased after stimulus onset; the reverse was true for alpha oscillatory peaks.*

SUPPLEMENTARY FIGURE 4

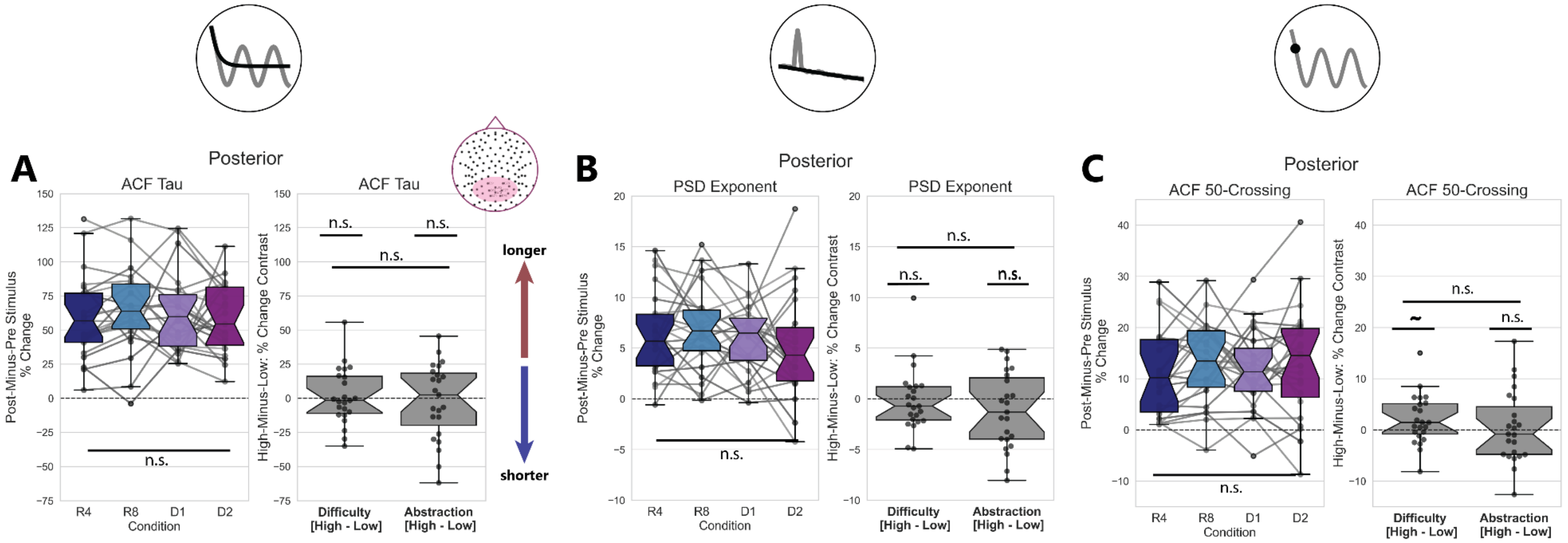

Figure S4: Aperiodic and oscillatory timescales lengthening from pre-to-post stimulus replicated in independent dataset

A) *Though it increases overall post-stimulus, the ACF Tau parameter measured at Posterior electrodes does not exhibit a condition-specific increase pre-to-post stimulus. This finding is similar to that of Dataset 1.*

B) *The Posterior PSD Exponent did increase post-stimulus relative to pre-stimulus, but this increase did not vary significantly across conditions.*

C) *The Posterior 50-Crossing measure from the ACFs of Dataset 2 did not show a main effect of condition, unlike the findings of Dataset 1. These data did exhibit a trending but non-significant effect of Difficulty.*
